# Can Soil Seed Banks Serve as Genetic Memory? A Study of Three Species with Contrasting Life History Strategies

**DOI:** 10.1371/journal.pone.0049471

**Published:** 2012-11-21

**Authors:** Bohumil Mandák, Petr Zákravský, Václav Mahelka, Ivana Plačková

**Affiliations:** 1 Institute of Botany, Academy of Sciences of the Czech Republic, Průhonice, Czech Republic; 2 Faculty of Environmental Sciences, Czech University of Life Sciences Prague, Prague, Czech Republic; CNR, Italy

## Abstract

We attempted to confirm that seed banks can be viewed as an important genetic reservoir by testing the hypothesis that standing (aboveground) plants represent a nonrandom sample of the seed bank. We sampled multilocus allozyme genotypes from three species with different life history strategies: *Amaranthus retroflexus, Carduus acanthoides*, *Pastinaca sativa*. In four populations of each species we analysed the extent to which allele and genotype frequencies vary in consecutive life history stages including the summer seed bank, which has been overlooked up to now. We compared the winter seed bank (i.e., seeds collected before the spring germination peak), seedlings, rosettes, the summer seed bank (i.e., seeds collected after the spring germination peak) and fruiting plants. We found that: (1) All three species partitioned most of their genetic diversity within life history stages and less among stages within populations and among populations. (2) All genetic diversity parameters, except for allele frequencies, were similar among all life history stages across all populations in different species. (3) There were differences in allele frequencies among life history stages at all localities in *Amaranthus retroflexus* and at three localities in both *Carduus acanthoides* and *Pastinaca sativa*. (4) Allele frequencies did not differ between the winter and summer seed bank in most *Carduus acanthoides* and *Pastinaca sativa* populations, but there was a marked difference in *Amaranthus retroflexus*. In conclusion, we have shown that the summer seed bank is not genetically depleted by spring germination and that a majority of genetic diversity remains in the soil through summer. We suggest that seed banks in the species investigated play an important role by *maintaining* genetic diversity sufficient for recovery rather than by *accumulating* new genetic diversity at each locality.

## Introduction

The term “seed bank” refers to dormant seeds that are present in the soil. Buried seeds of many species can retain their viability for long periods [1], [2]. New individuals are recruited from the seed bank over time, which may strongly affect vegetation composition, structure and dynamics [3]. In general, there are two types of seed banks: transient and persistent [3], [4]. Transient seed banks are composed of seeds that germinate within a year of their initial dispersal and may not be viable at the onset of the next growing season. Persistent seed banks are made up of seeds that remain viable in the soil for longer than one year. While many plants develop vegetatively, some species depend on seed reserves that have accumulated in the soil between disturbances. Thompson and Grime [5], who studied ten contrasting habitats, found that at every site there were some species which had a persistent seed bank and some whose seeds were present for only part of the year. The size of the buried seed pool reflects the type, intensity and frequency of disturbance [6] and can significantly differ among species. Over the past 20 years, however, a growing volume of literature dealing with buried seeds has mainly focused on the importance of seed banks for population recovery following disturbances of established vegetation, that is, from an ecological point of view [3], [4], [6], [7].

Besides the ecological role of seed banks, several authors have studied their importance from an evolutionary perspective. This idea was first formulated by Templeton and Levin [8]. In their view, seed banks can serve as (i) regeneration pools with reasonable genetic variability that may be an important determinant of the success of different species at a locality, buffering the effects of local extinction of genotypes in adult populations caused by selection or drift, or (ii) as an “evolutionary memory” which stores genotypes for a variable number of years [9], possibly slowing the rate of evolutionary change [8], [10].

Many authors have tried to confirm that seed banks represent important genetic reservoirs by testing the hypothesis that standing (aboveground) plants represent a nonrandom sample of their species’ seed bank [11]–[15]. If seed banks really function as genetic reservoirs by maintaining genetic diversity, they must be genetically more diverse than subsequent life history stages due to accumulation of different genotypes over time. The presence of diverse genotypes in soil seed banks may then serve as raw material for evolutionary processes that are behind the transformation of soil seeds into reproductive populations. Despite their effort, scientists have been unable to conclusively confirm such a role of seed banks in evolutionary dynamics of plant populations [9], [11]–[17]. While some authors have demonstrated differences between seed banks and aboveground populations such as higher genetic diversity of seed bank populations [9], [13], higher heterozygosity of aboveground populations [11]–[15] or significant differences in allele frequencies [11], [12], [14], [15], studies of others show no differences in any of the population genetic parameters investigated [16], [17]. This discrepancy stems partly from different methodological approaches to comparing genetic diversity parameters of seed banks with the parameters of different stages of aboveground populations (i.e., seedlings or adults, or combination of both). Most importantly, ignoring the seedling stage could lead to incorrect interpretations of differences between seed banks and adult populations, as such differences may be due to microselective forces acting on early aboveground populations, rather than due to storing genetic variability in the soil or germination and establishment success [15].

Three mechanisms have been proposed to explain the commonly observed pattern of higher homozygosity and inbreeding in seed banks [9], [11]–[16], [18], [19]. First, it could be explained by higher inbreeding in past years, as argued by Tonsor *et al*. [14]. Secondly, it could be attributed to a temporal Wahlund effect [14], but see [20]. Thirdly, Vitalis *et al*. [20] point out that the most probable mechanism responsible is natural selection, which progressively eliminates less fit homozygotes through, for example, their lower germinability [21], [22] or a strong self-thinning process between the seedling and mature plant stage, as described by Mandák *et al*. [15].

**Table 1:** Analysis of molecular variance in *Amaranthus retroflexus, Carduus acanthoides* and *Pastinaca sativa*.

Source of variation	d.f.	Sum of squares	Variance component	Fixation index	Percentage of variance
*Amaranthus retroflexus*					
Among populations	3	161.56	0.17*	0.29	18.23
Among stages within populations	12	86.49	0.10*	0.13	10.58
Within stages	1106	714.55	0.65*	0.18	71.19
Total	1121	962.59	0.91		
*Carduus acanthoides*					
Among populations	3	115.63	0.09*	0.07	5.74
Among stages within populations	12	32.78	0.01*	0.01	0.82
Within stages	1502	2296.77	1.53*	0.06	93.44
Total	1517	2445.18	1.64		
*Pastinaca sativa*					
Among populations	3	40.93	0.03*	0.03	3.03
Among stages within populations	16	33.74	0.02*	0.01	1.42
Within stages	1312	1414.32	1.08*	0.04	95.55
Total	1331	1488.98	1.13		

Analysis of molecular variance among populations, among life history stages within populations, and within life history stages of *Amaranthus retroflexus*, *Carduus acanthoides* and *Pastinaca sativa*. Significance of variance components were tested by a permutation test (*P<0.000001).

Only a few empirical studies have investigated the genetic relationship between above- and belowground populations on large data sets (i.e., from more than five localities of a single species). Moreover, few studies (e.g., [12], [17], [23]) have investigated the genetic relationship between seed banks and surface plants across large spatial scales. Cabin *et al*. [12] found differences between above- and belowground populations at four out of five localities of *Lesquerella fendleri*. By contrast, Lundemo *et al*. [23] and Falahati-Anbaran *et al*. [17] did not find any differences in gene diversity for *Arabidopsis thaliana* and *Arabidopsis lyrata* subsp. *petraea*, respectively. Such large-scale, multipopulation studies are necessary to test whether there are any broad genetic differences between soil seeds and surface plants rather than mere idiosyncratic differences between these life-history stages in particular populations. These studies will also allow to test the hypothesis that seed banks reduce the rate of population differentiation by retaining alleles preserved from different generations [13], [14].

In this study, we have introduced the term “summer seed bank”. It refers to seeds that persist in the soil after the spring germination peak and before new seeds are added to the soil by fruiting plants. It represents the actual seed bank for the next year(s) even if the aboveground population is completely destroyed. The summer seed bank thus holds very important information concerning the real amount of genetic diversity persisting in the seed bank through summer. If we compare only genetic diversity in the soil seed bank with the diversity of standing plants, we completely miss any information concerning genetic diversity that is actually present in the seed bank through summer. Despite this, the role of the summer seed bank has been overlooked until now, and studies that deal with the genetic relationship among various life history stages, including the summer seed bank on larger spatial scales, are missing. Such studies may answer the question: “What factors are responsible for nonrandom patterns of genetic variability commonly found in natural populations?” In this study, we sampled multilocus allozyme genotypes from three species with different life history strategies to test whether there are any broad genetic differences between soil seeds and surface plants. We analysed four populations of each species to ascertain the extent to which allele and genotype frequencies vary among life history stages including the summer seed bank. Specifically, we sought answers to the following questions: (1) Are there any differences in population genetic characteristics among species with different life history strategies? (2) Is the pattern of genetic variability consistent over several localities within each species? (3) Does the winter and summer seed bank differ from other life history stages and do seed banks in general thus serve as a genetic reservoir of the population? (4) Do seed banks reduce the rate of among-population differentiation?

**Table 2:** Population genetic characteristics for individual life history stages of *Amaranthus retroflexus*, *Carduus acanthoides* and *Pastinaca sativa*.

Diversity measure	WSB	Seedlings	Rosette	SSB	Fruiting plants	*P*
*Amaranthus retroflexus*						
*R*_S_	1.787	1.736	–	1.780	1.668	0.821
*H*_O_	0.076	0.107	–	0.096	0.100	0.847
*H*_S_	0.222	0.230	–	0.210	0.207	0.941
*F*_IS_	0.658*	0.533*	–	0.543*	0.516*	0.457
*F*_ST_	0.287*	0.244*	–	0.345*	0.256*	0.938
*Carduus acanthoides*						
*R*_S_	2.161	–	2.141	2.026	2.214	0.580
*H*_O_	0.339	–	0.338	0.346	0.378	0.262
*H*_S_	0.380	–	0.386	0.367	0.399	0.451
*F*_IS_	0.107*	–	0.125*	0.055*	0.053*	0.244
*F*_ST_	0.059*	–	0.082*	0.052*	0.056*	0.386
*Pastinaca sativa*						
*R*_S_	1.763	1.746	1.764	1.582	1.754	0.153
*H*_O_	0.284	0.295	0.286	0.271	0.292	0.899
*H*_S_	0.279	0.264	0.274	0.238	0.270	0.454
*F*_IS_	–0.017	–0.118	–0.041	–0.141	–0.081	0.173
*F*_ST_	0.093*	0.023*	0.023*	0.035*	0.047*	0.382

Statistical comparison of allelic richness (*R*_S_), observed heterozygosity (*H*_O_), gene diversity (*H*_S_), inbreeding coefficient (*F*_IS_) and levels of differentiation among populations (*F*_ST_) for individual life history stages of *Amaranthus retroflexus*, *Carduus acanthoides* and *Pastinaca sativa*. WSB – winter seed bank, SSB – summer seed bank. Probability values for differences between life history stages are for two-sided *t*-tests after 10 000 permutations. * Significant deviation (P<0.05) from the null expectation of *F = *0. The analyses were performed using FSTAT software [47].

## Results

### Hierarchical Analysis of Molecular Variance

Hierarchical analyses of molecular variance (AMOVA) computed separately for *Amaranthus retroflexus*, *Carduus acanthoides* and *Pastinaca sativa* revealed that most of the genetic diversity was partitioned within life history stages; the analysis accounted for 71.19, 93.44 and 95.55% of the overall variance, respectively (Table 1). In *Carduus acanthoides* and *Pastinaca sativa*, only 0.82 and 1.42% of the genetic diversity was partitioned among stages within populations, and 5.74 and 3.03% was partitioned among populations, respectively. *Amaranthus retroflexus,* on the other hand, showed higher differentiation among populations as well as among stages within populations (Table 1).

**Figure 1:**
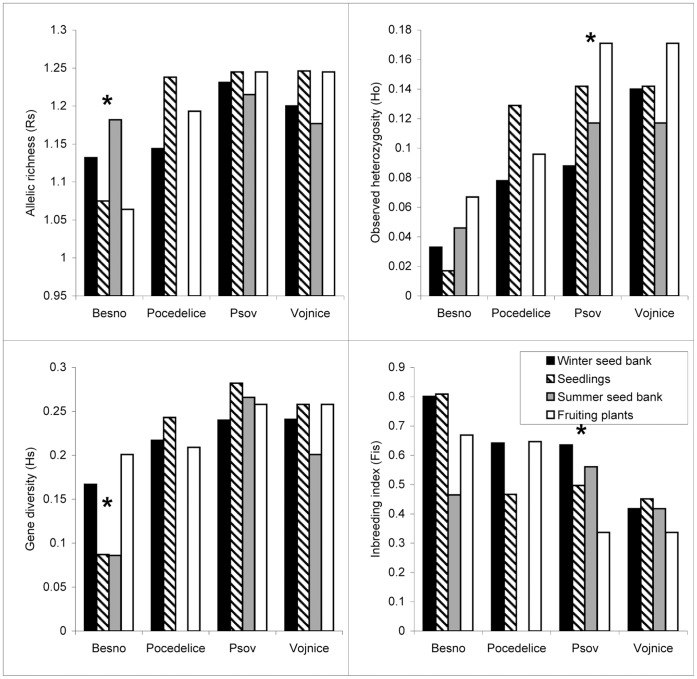
Population genetic characteristics of *Amaranthus retroflexus*. Comparison of allelic richness (*R*_S_), observed heterozygosity (*H*_O_), gene diversity (*H*_S_) and inbreeding coefficient (*F*_IS_) for individual life history stages of *Amaranthus retroflexus* at four localities. * Significant differences among life history stages within a locality were identified by two-sided *t*-tests after 10 000 permutations (analysis performed using FSTAT [47]).

**Figure 2:**
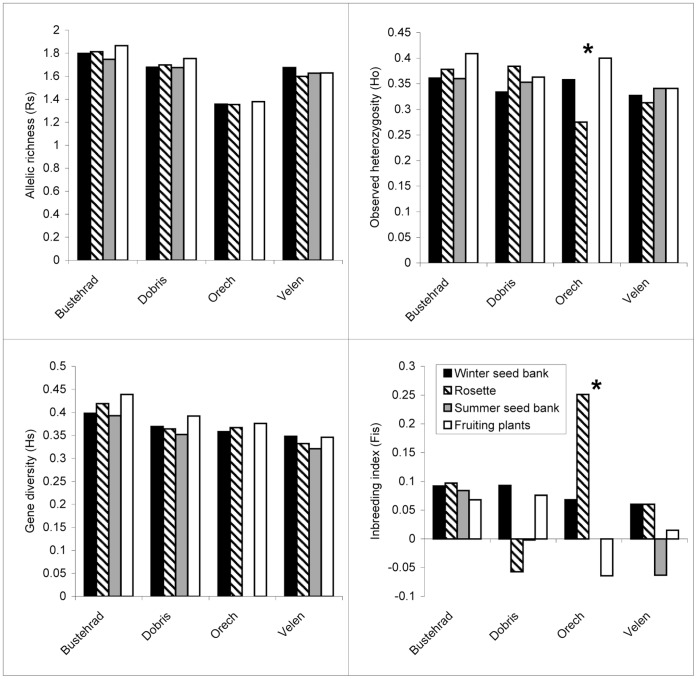
Population genetic characteristics of *Carduus acanthoides*. Comparison of allelic richness (*R*_S_), observed heterozygosity (*H*_O_), gene diversity (*H*_S_) and inbreeding coefficient (*F*_IS_) for individual life history stages of *Carduus acanthoides* at four localities. * Significant differences among life history stages within a locality were determined by two-sided *t*-tests after 10 000 permutations (analysis performed using FSTAT [47]).

**Figure 3:**
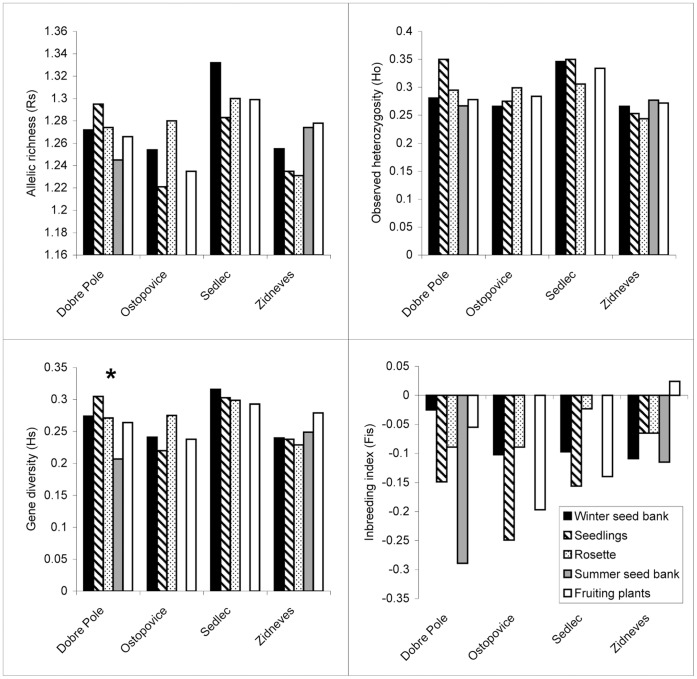
Population genetic characteristics of *Pastinaca sativa*. Comparison of allelic richness (*R*_S_), observed heterozygosity (*H*_O_), gene diversity (*H*_S_) and inbreeding coefficient (*F*_IS_) for individual life history stages of *Pastinaca sativa* at four localities. * Significant differences among life history stages within a locality were determined by two-sided *t*-tests after 10 000 permutations (analysis performed using FSTAT [47]).

### Comparison of Life History Stages Across Populations

When we compared population genetic characteristics – i.e., allelic richness (*R*_S_), observed heterozygosity (*H*_O_), gene diversity (*H*_S_), inbreeding coefficient *f*(*F*_IS_) and levels of differentiation among populations *θ*(*F*_ST_) – among life history stages across all populations of individual species, we did not reveal any statistically significant differences (Table 2). Moreover, inbreeding coefficients *f*(*F*_IS_) in *Amaranthus retroflexus* and *Carduus acanthoides*, and fixation indices *θ*(*F*_ST_) in all species and life history stages were significantly different from zero (Table 2).

**Table 3:** Tests for differences in allele frequencies of individual loci and over all loci.

Locus	Locality			
*Amaranthus retroflexus*	Besno	Pocedelice	Psov	Vojnice
6-Pgdh-1	–	–	–	1.000
6-Pgdh-2	–	–	–	–
Pgm	0.060	0.105	**0.001**	0.057
Dia-2	**0.029**	**0.042**	0.212	0.338
Est-1	**<10^–4^**	**<10^–4^**	**<10^–4^**	**<10^–4^**
Adh	–	–	–	–
Over loci	**<10^–4^**	**<10^–4^**	**<10^–4^**	**0.001**
*Carduus acanthoides*	Orech	Bustehrad	Dobris	Velen
Lap	0.831	0.400	0.683	0.987
Shdh	**<10^–4^**	**<10^–4^**	**<10^–4^**	**0.020**
Adh	0.890	0.126	0.928	0.594
6-Pgdh-1	0.964	0.089	**0.009**	0.702
6-Pgdh-2	0.431	0.900	0.755	0.534
Aat-1	0.538	0.138	**0.008**	0.957
Aat-2	**0.019**	0.070	0.109	0.063
Dia-2	**0.020**	0.060	**0.012**	0.963
Over loci	**0.003**	**0.0001**	**<10^–4^**	0.415
*Pastinaca sativa*	Dobre Pole	Ostopovice	Sedlec	Zidneves
Lap	0.218	**0.032**	0.096	0.161
Shdh	0.889	0.356	0.871	0.095
Nadh-Dh	0.490	**0.012**	1.000	0.406
6-Pgdh-1	1.000	1.000	1.000	1.000
Aat-1	0.773	0.493	0.440	–
Aat-2	0.274	0.241	0.401	–
Pgm	**0.005**	0.211	**<10^–4^**	–
Est	**0.008**	**0.068**	**0.005**	0.147
Over loci	**0.033**	**0.015**	**0.001**	0.174

Probabilities for a comparison of individual loci and over all loci at four localities computed by exact tests for population differentiation [50] to determine if there are significant differences in allele frequencies among life history stages of *Amaranthus retroflexus*, *Carduus acanthoides* and *Pastinaca sativa*. The analysis was carried out using TFPGA software [51]. – only one allele at the locus, i.e. the analysis was not performed.

### Comparison of Life History Stages within Populations

When we tested differences among life history stages separately for individual populations of the three species, we obtained inconsistent results concerning individual parameters, populations and species (Figs. 1, 2, 3). We found significant difference only for *H*_O_ and *f*(*F*_IS_) in *Carduus acanthoides* at one locality (locality Orech) (Fig. 2) and only for *H*_S_ in *Pastinaca sativa* at one locality (Fig. 3). Statistically significant differences detected in *Amaranthus retroflexus* were not consistent over all sampled populations (Fig. 1). Significant differences in *R*_S_ and *H*_S_ were found at the locality Besno, but not at the other localities. In the same way, observed heterozygosity (*H*_O_) and inbreeding coefficient *f*(*F*_IS_) were significantly different among life history stages only at locality Psov.

Although allele frequencies differed among life history stages at all localities in *Amaranthus retroflexus*, the results for both *Carduus acanthoides* and *Pastinaca sativa* were not consistent (Table 3). Table 3 also shows the influence of particular loci on the results computed over all loci. Allele frequencies are usually significantly different only at some specific loci over all localities. For *Amaranthus retroflexus*, it is locus Est-1 and in some cases Dia-2. In *Carduus acanthoides* Shdh participates importantly with a lower contribution of other loci. The Est locus and in some cases the Pgm locus are important for differentiating among life history stages in *Pastinaca sativa* (Table 3).

**Table 4:** Tests for differences in allele frequencies among individual life history stages of *Carduus acanthoides*.

locality Dobris	Winter seed bank	Rosettes	Summer seed bank
Winter seed bank	–		
Rosettes	**<10^–4^**	–	
Summer seed bank	0.3673	**0.0178**	–
Fruiting plants	**0.0124**	0.4107	0.1029
**locality Bustehrad**			
Winter seed bank	–		
Rosettes	0.3356	–	
Summer seed bank	**0.0175**	**0.0021**	–
Fruiting plants	**0.0095**	0.1619	**0.0039**
**locality Orech**			
Winter seed bank	–		
Rosettes	**0.0047**	–	
Summer seed bank	0.1547	**0.0178**	–
Fruiting plants	0.1107	0.9772	0.2390
**locality Velen**			
Winter seed bank	–		
Rosettes	0.2081	–	
Summer seed bank	0.3570	0.6022	–
Fruiting plants	0.9512	0.9822	0.7812

Matrix of combined probabilities for each pairwise comparison over all loci computed by exact tests for population differentiation [50] is presented. Probabilities are given for different localities. The analysis was carried out using TFPGA software [51].

**Table 5:** Tests for differences in allele frequencies among individual life history stages of *Amaranthus retroflexus*.

locality Besno	Winter seed bank	Seedlings	Summer seed bank
Winter seed bank	–		
Seedlings	**0.0042**	–	
Summer seed bank	**0.0030**	**0.0001**	–
Fruiting plants	**0.0001**	**0.0020**	**<10^–4^**
**locality Pocedelice**			
Winter seed bank	–		
Seedlings	**0.0100**	–	
Summer seed bank	**0.0004**	**<10^–4^**	–
Fruiting plants	**0.0010**	**0.0002**	**0.0001**
**locality Psov**			
Winter seed bank	–		
Seedlings	0.0944	–	
Summer seed bank	**<10^–4^**	**0.0001**	–
Fruiting plants	**0.0003**	**0.0021**	**<10^–4^**
**locality Vojnice**			
Winter seed bank	–		
Seedlings	0.3279	–	
Summer seed bank	**0.0027**	**0.0025**	–
Fruiting plants	0.3102	**0.0417**	**0.0009**

Matrix of combined probabilities for each pairwise comparison over all loci computed by exact tests for population differentiation [50] is presented. Probabilities are given for different localities. The analysis was carried out using TFPGA software [51].

**Table 6:** Tests for differences in allele frequencies among individual life history stages of *Pastinaca sativa*.

locality Sedlec	Winter seed bank	Seedlings	Rosettes	Summer seed bank
Winter seed bank	–			
Seedlings	**0.0128**	–		
Rosettes	**<10^–4^**	0.6726	–	
Summer seed bank	0.1966	0.9967	0.9851	–
Fruiting plants	**0.0052**	0.9956	0.9046	0.9976
**locality Dobre Pole**				
Winter seed bank	–			
Seedlings	0.3229	–		
Rosettes	0.4072	0.9966	–	
Summer seed bank	0.1585	0.7474	0.7659	–
Fruiting plants	0.1408	0.5301	0.2298	0.7356
**locality Ostopovice**				
Winter seed bank	–			
Seedlings	0.3645	–		
Rosettes	0.0856	0.7317	–	
Summer seed bank	0.9849	0.9666	0.9915	–
Fruiting plants	**0.0017**	0.8502	0.4812	0.8583
**locality Zidneves**				
Winter seed bank	–			
Seedlings	0.4815	–		
Rosettes	0.1444	0.8778	–	
Summer seed bank	0.5794	0.9363	0.5010	–
Fruiting plants	0.2908	0.4777	0.3211	0.2546

Matrix of combined probabilities for each pairwise comparison over all loci computed by exact tests for population differentiation [50] is presented. Probabilities are given for different localities. The analysis was carried out using TFPGA software [51].

To understand changes in allele frequencies further, we tested allele frequencies separately for each species and locality (Tables 4, 5, 6). Significant differences in allele frequencies were most often encountered in *Amaranthus retroflexus*, followed by *Carduus acanthoides* and *Pastinaca sativa*. The results are very diverse even within species. In other words, there are populations with stages completely different among each other (e.g., Besno and Pocedelice in *Amaranthus*, Table 5) as well as populations with similar allele frequencies over all stages (e.g., Dobre Pole and Zidneves in *Pastinaca*; Table 6).

However, from simple allele presence/absence counting within individual life history stages, it is evident that, in *Amaranthus retroflexus,* only in two cases out of 64 was an allele present in the summer seed bank and not in other stages; in no case was an allele detected only in the winter seed bank (Table S1). *Carduus acanthoides* did not allocate a significantly higher proportion of genetic diversity to its winter or summer seed bank than to its aboveground populations. Only in five cases out of 100 (Table S2) did we detect alleles present only in the seed bank and absent from any other stage. *Pastinaca sativa*, exhibited a similar pattern as *Carduus acanthoides*, i.e. only in two cases out of 120, the allele was present in the winter seed bank and absent from other stages (Table S3).

**Table 7:** Genetic diversity of *Amaranthus retroflexus*, *Carduus acanthoides* and *Pastinaca sativa* (from other studies).

Genetic diversity	*Amaranthus retroflexus*	*Carduus acanthoides*	*Pastinaca sativa*
*PL*	50.0	84.5	85.7
*A*	2.01	2.37	1.92
*H*_o_	0.142	0.330	0.295
*H*_e_	0.227	0.364	0.336
*F*(*F*_IT_)	0.548*	0.173*	0.130*
*f*(*F*_IS_)	0.382*	0.097*	–0.161
*θ*(*F*_ST_)	0.270*	0.085*	0.251*

Comparison of genetic diversity within 20 populations of *Amaranthus retroflexus*, *Carduus acanthoides* and *Pastinaca sativa* based on allozyme loci (data taken from [45], [46] and Mandák *et al*., unpublished data, respectively). *PL* = percentage of polymorphic loci. *A* = average number of alleles per polymorphic locus. *H*_o_ = observed heterozygosity. *H*_e_ = expected heterozygosity. *F*, *f*, *θ* = Weir and Cockerham’s estimates of Wright’s *F* statistics (*F*_IT_, *F*_IS_ and *F*_ST_, respectively) which represents deviations from Hardy-Weinberg expectations over all populations, deviations within individual populations and the proportion of total genetic diversity partitioned among populations. * Significant deviation (P<0.05) from the null expectation of *F = *0. The analysis was performed using FSTAT software [47].

## Discussion

The potential role of soil seed banks in evolutionary dynamics of plant populations has evoked many theoretical [20], [24]–[31] as well as empirical studies [9], [11]–[17], [32]–[35]. Some of these studies view seed banks as important genetic reservoirs that affect the evolution and dynamics of aboveground populations. If seed banks were true genetic reservoirs, they should accumulate genotypes over time and would have the effect of slowing down population differentiation by retaining alleles preserved over multiple generations. Our study, contrary to most published results, does not support the hypothesis that seed banks contain significantly larger amounts of genetic diversity than other life history stages. The patterns of partitioning of genetic diversity were similar in all three species. *Amaranthus retroflexus*, however, partitioned a higher amount of molecular variance among populations and among stages within populations compared to the other two species (Table 1). The annual invasive *Amaranthus retroflexus* is predominantly a selfing species. It is therefore not surprising that it forms genetically differentiated populations. Besides this, it has the highest reproductive output, and its seedling populations are the densest, so self-thinning can alter them substantially, leading to differentiation among stages (see also [15] for *Atriplex tatarica*). As pointed out by Morris et al. [9], the capacity of seed banks to retain higher levels of genetic diversity may depend on (1) seed dormancy characteristics and potential multigenerational contributions to the seed bank, and (2) the relative size of seed bank populations. Species producing large seed banks with dormant seeds would be expected to contain higher levels of genetic diversity in their seed banks in comparison to other life history stages [8], [9], [13]. Among our study species, it seems that mainly *Amaranthus retroflexus* has the potential to build large, persistent seed banks. This species, however, did not accumulate higher genetic diversity in its seed bank either. The winter seed bank did not accumulate genetic diversity and was not significantly different from other stages in other species such as *Calluna vulgaris* [16], *Atriplex tatarica* [15], *Cardamine amara* [19] and *Arabidopsis lyrata* subsp. *petraea* [17]. Honnay *et al*. [36], in their meta-analysis of 13 published studies comparing genetic diversity between the seed bank and other life history stages, found no evidence of high levels of genetic diversity accumulating in the soil seed bank. However, differences in allele frequencies between the seed bank and aboveground plants were detected in almost all cases [36]. They noted, like Vitalis *et al*. [20] or Mandák *et al*. [15] before, that “if genetic differences are present between the standing crop and the seed bank, they are very likely the result of local selection acting either directly or indirectly as a filter on the alleles present in the seed bank”. We also found differences in allele frequencies in all species and at all but two localities (Table 3). However, consistent differences among individual life history stages were only encountered in *Amaranthus* (Tables 4, 5, 6). We observed the same trend when we compared allele frequencies between the winter seed bank and the summer seed bank (Tables 4, 5, 6). The changes in allele frequencies in *Amaranthus* contrast with the other population genetic characteristics, in which no significant differences among individual life history stages were observed (Table 2). These results suggest that while spring germination does not genetically deplete the seed bank when most of the genetic diversity is maintained in the soil through summer, forces affecting allele frequencies such as natural selection or drift (or a combination of both) may play a more important role in shaping the populations of *Amaranthus* through their maturation process (see also [15] for *Atriplex tatarica*). In the annual and invasive *Amaranthus retroflexus,* the number of seeds in the seed bank must decrease dramatically due to massive spring germination. The process of self-thinning can later remove a significant portion of individuals, which might be the reason why changes in allele frequencies were observed among nearly all life history stages. In the perennial, outcrossing species, this question seems less relevant. However, the analysis of Honnay et al. [36] does not confirm this hypothesis entirely since by far the most of their investigated species were perennials. One possible explanation for this discrepancy is that different stages of aboveground populations were investigated, i.e., seedlings, small immature plants, mature plants or fruiting plants, in our and in the other studies (see also [12], [15]).

**Figure 4:**
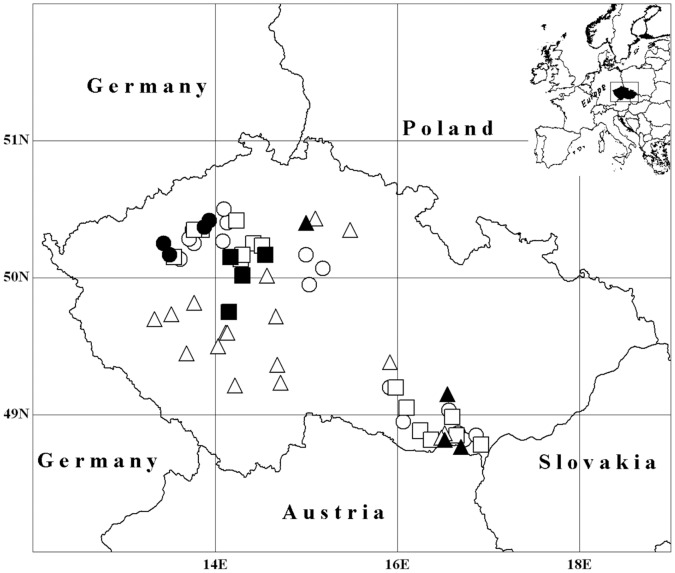
Location of *Amaranthus retroflexus, Carduus acanthoides* and *Pastinaca sativa* populations. Map showing the location of the 20 populations of each of the three species under study, i.e. *Amaranthus retroflexus* (empty circles), *Carduus acanthoides* (empty squares) and *Pastinaca sativa* (empty triangles). Four localities of each species were selected and used in the present study (highlighted by full symbols).

**Table 8:** Summary of sampled localities of individual species.

Locality	Latitude (N)	Longitude (E)	Localization	Site condition
*Amaranthus retroflexus*				
Besno	50°10′59′′	13°30′25′′	Běsno, on the SW margine of the village	Hop garden
Pocedelice	50°22′17′′	13°53′03′′	Počedělice, on the W margin of the village	Hop garden
Psov	50°15′23′′	13°26′47′′	Pšov, ca 0.5 km W of the village	Hop garden
Vojnice	50°25′05′′	13°56′19′′	Vojnice, ca 0.5 km S of the village	Hop garden
*Carduus acanthoides*				
Bustehrad	50°09′42′′	14°10′53′′	Buštěhrad, ca 1.5 km SW of the village	Abandoned dumping ground, open community dominated by annual species
Dobris	49°45′56′′	14°09′56′′	Dobříš, ca 1.6 km S of the village	Abandoned field, sparse grassland
Orech	50°01′14′′	14°18′13′′	Ořech, on the E margin of the village	Abandoned field, grassland
Velen	50°10′35′′	14°33′39′′	Veleň, on the E margin of the village	Mown sparse grassland
*Pastinaca sativa*				
Dobre Pole	48°49′20′′	16°31′50′′	Dobré Pole, on the SW margin of the village	Margin of salt meadow
Ostopovice	49°10′02′′	16°32′32′′	Ostopovice, on the S margin of the village	Periodically mown meadow
Sedlec	48°46′27′′	16°42′14′′	Sedlec, ca 0.8 km SE of the village	Margin of salt meadow
Zidneves	50°24′55′′	15°00′06′′	Židněves, on the NE margin of the village	Periodically mown meadow

Even though the presence of a seed bank acting as a long-term reservoir of genetic diversity has been first pondered by Templeton & Levin [8] and later supported by quite a large number of empirical studies, experiments covering multiple species and multiple populations of each species with the summer seed bank included in the experiments do not support this idea unequivocally. In our view, the question whether seed banks differ from standing aboveground populations should be replaced by the question whether the winter seed bank differs from the summer seed bank, which represents the actual genetic reserve for the following years. The winter seed bank is a sum of genetic diversity accumulated over years due to, for example, selective germination [11], [22]. It also contains genetic diversity that is added each year in the form of seeds falling from fruiting plants [37]. On the other hand, the summer seed bank contains seeds that did not germinate in spring, and thus may store genotypes for which the conditions of the particular environment or season are not favorable, or remain dormant. Hence, by comparing the winter seed bank with aboveground populations, we cannot draw a conclusion regarding the seed bank as a possible reservoir of genetic diversity because we do not know what really remains in it. In the present study, the overall genetic diversity stored in the winter seed bank was similar to the one that actually remained in the summer seed bank and other life history stages. From this point of view, the seed bank can serve as a genetic reservoir by maintaining, but not accumulating, genetic diversity. We therefore cannot state that seed banks play an important role in *accumulating* local genetic diversity. Instead, our results suggest that seed banks in the species investigated play an important role by merely *maintaining* genetic diversity sufficient for recovery of population genetic diversity at localities.

### Conclusions

We have demonstrated for the first time the extent to which the summer soil seed bank differs from other life history stages. Knowing this difference is crucial for answering the question that concerns the soil seed bank as a genetic reservoir of a population. We have clearly shown that the summer seed bank is not completely depleted by spring germination and that most of the genetic diversity can survive through summer. Our results support the assumption that seed banks may protect plant populations from detrimental consequences of size fluctuations. We suggest that seed banks in the species investigated play an important role by *maintaining* genetic diversity sufficient for recovery rather than by *accumulating* new genetic diversity at each locality. Finally, we have shown that seed banks in the species under study did not have the effect of slowing population differentiation by retaining alleles preserved over multiple generations.

## Materials and Methods

### Study Species

We chose the following species for our study: *Amaranthus retroflexus* L., *Carduus acanthoides* L. and *Pastinaca sativa* L. subsp. *sativa*. The principal criteria for species selection were diploid ploidy level and production of persistent seed banks. Furthermore, we selected species with different life history strategies and habitat preferences in order to test whether there are any broad genetic differences. The species characteristics were mostly taken from [38] for *Amaranthus*, [39] for *Pastinaca* and [40] for *Carduus* and can be summarized as follows: *Amaranthus retroflexus* is an annual species of highly disturbed habitats. It is a neophyte to the Czech flora, with its first record of occurrence from 1818 [41]. *Amaranthus retroflexus* is a self-compatible and probably primarily self-pollinated species, able to produce huge numbers of seeds, depending on the growing conditions. Seed production varies between tens and hundreds of thousands and occasionally reaches millions of seeds per plant. Seeds with impermeable and both mechanically and chemically resistant coats form persistent seed banks. Buried seeds can remain viable for at least 6 to 10 years. This period, however, may vary depending on the depth of burial, soil temperature and moisture, frequency of soil disturbance, and dormancy. Dormancy is regulated primarily by seasonal variation in temperature. Small seeds and a structured seed coat facilitate dispersal by water, animals (mainly humans) and, to a lesser extent, by wind.

*Carduus acanthoides* is an annual, winter annual or biennial species of moderately disturbed habitats. It is an archaeophyte in the Czech flora [41]. It is primarily an outcrossing species, but considerable variation in the degree of selfing among individuals was recorded. Flowers are pollinated by insects. Seed production varies with climatic conditions and ranges between hundreds and thousands of seeds per head. It reproduces strictly by seeds, which can be dispersed by wind using the attached pappus. The seeds fall within 50 m of the maternal plant, but less than l percent of seeds may be carried further than 100 m apart. Seed viability remains high over several years. *Carduus acanthoides* survives winter either as seeds or rosettes. The species forms dense stands at disturbed sites where competition is low.

*Pastinaca sativa* is a biennial or short-lived, monocarpic, perennial species of habitats with a low level of disturbance [42], [43]. It is native to the flora of the Czech Republic [41]. Mostly hermaphroditic flowers of *P. sativa* are protandrous, promoting outcrossing between different plants. The species is insect-pollinated and able to produce up to 2000 fruits per plant. Its seeds are efficiently dispersed by wind to a distance of several meters from the maternal plant as well as by humans along roadways and highways. The seeds of *P. sativa* possess morphological dormancy due to an underdeveloped embryo. Most seeds therefore germinate in the following spring [44]. Seeds of this species only form a weak, persistent seed bank; 99 percent of all seedlings typically emerge within 2 years after seed dispersal ([39] and references therein). After emergence, seedlings develop a strong taproot for nutrient storage and overwintering as rosettes.

### The Species’ Genetic Diversity

Population genetic diversity of the three species, regardless of their different life history stages, has been investigated earlier. A summary of population genetic diversity across 20 populations of *Amaranthus retroflexus* and *Carduus acanthoides* has been published by Mandák *et al*. ([45], [46], respectively). Population genetic data for *Pastinaca sativa* has not been published so far, but the sampling strategy (20 localities and 15 plants regularly sampled per locality) was same as in Mandák *et al*. [45], [46] (see Table 7 for overview of genetic diversity of individual species). The three species strongly differed in their population genetic characteristics. The most genetically impoverished species was the annual invasive species *Amaranthus retroflexus* for which high inbreeding and among population differentiation was indicated (Table 7). However, Mandák *et al.* [45] demonstrated that *A. retroflexus* supports a larger amount of genetic variation than other widespread selfing species. Although both *Carduus acanthoides* and *Pastinaca sativa* exhibited low levels of inbreeding, populations of *Pastinaca sativa* were more structured than those of *Carduus acanthoides* (Table 7). The low *θ*(*F*_ST_) value is obviously the result of the high dispersal capacity of *Carduus acanthoides* [46] in comparison to *Pastinaca sativa*, which lacks any apparatus that would enable dispersal over long distances. In the present study, we therefore compared individual life history stages of species that differ not only in their life history strategies but also in levels and patterns of genetic diversity in order to test whether there are any broad genetic differences between soil seeds and surface plants.

### Study Sites

We tested the relationship among plants derived from seeds extracted from the soil seed bank, seedlings, rosettes and mature plants over a range of localities and species. We took samples at four different localities of our species’ ranges. Populations were selected on the basis of known genetic variability (see above), i.e. the most genetically variable populations were selected (Fig. 4). These differed in habitat dynamics and population size or history (see Table 8, Fig. 4). This minimized the risk that our data would be distorted by attributes of one particular locality.

### Sample Collection and Allozyme Analysis

In four natural populations of our three species, we took 40 samples of each life history stage from regularly distributed permanent plots. To ensure sampling consistency, we used the same sampling strategy for all three species. To obtain samples from different phases of the life history cycle for our genetic analysis, we sampled several consecutive life history stages: (1) the winter soil seed bank (i.e. seed bank before the spring germination peak), (2) seedlings, (3) rosettes, (4) the summer soil seed bank (i.e. seed bank after the spring germination peak) and (5) fruiting plants (i.e. plants that survived self-thinning as well as the competition from the surrounding vegetation, see also [15]). The number of sampled stages differed in relation to the species and its life history cycle (see Table 2). In the case of the annual species *Amaranthus retroflexus*, we did not sample the rosette life history stage. In the perennial *Carduus acanthoides*, we sampled only the rosette life history stage instead of the seedling stage because we were unable to differentiate between large seedlings and small rosettes in the field. In *Pastinaca sativa*, we were able to sample all stages because they strongly differed among each other. We did not succeed in obtaining the summer seed banks from four populations: Pocedelice (*Amaranthus*), Orech (*Carduus*), and Ostopovice and Sedlec (*Pastinaca*).

Soil samples of the winter seed bank were collected in February, of the summer seed bank in July. *Amaranthus retroflexus* was sampled in 2006, *Carduus acanthoides* in 2007 and *Pastinaca sativa* in 2008. First, we created a 15 by 10 m grid with gridlines spaced at 5 m intervals. We then took soil samples of winter seed bank at the intersections of the gridlines by digging up soil blocks that were 20 cm long, 20 cm wide and 10 cm deep. We recorded the spatial coordinates of each sample. Soil samples were moved to the Experimental garden of the Institute of Botany, Academy of Sciences, Průhonice, Czech Republic (49°59′30′′N, 14°34′00′′E, ca 320 m a.s.l.). Each soil block was slightly disturbed to increase the probability of seed germination and placed on a layer of sterilized soil in a 25×25×25 cm garden pot. Emerging seedlings were harvested and transplanted into small pots. After 14 days, the soil was disturbed once again and sprayed with a gibberellic acid solution to break dormancy and speed up as well as synchronise germination of ungerminated seeds. From each population, forty seedlings were randomly harvested, grown to maturity and subjected to allozyme electrophoresis. We believe that we were able to establish plants from most seeds stored in the seed banks. Using a gibberellic acid solution, we broke dormancy of a majority of ungerminated dormant seeds because only a minimal portion of the seeds remained in the soil. We have to admit, however, that our results could be influenced by a bias in the differential establishment rates of seeds from different seed banks collected from different populations and different species. The same problem was encountered by Mandák et al. for *Atriplex tatarica* [22]. They pointed out that this possibility is to some extent a technical issue because seedlings must grow large enough before they can be used for allozyme analysis, and many plants experience some degree of early mortality. On the other hand, the use of small seedlings is impossible because of the low enzyme activity in this life stage as found in *Atriplex tatarica*.

We then collected subsequent life history stages within the grid established during the winter seed bank soil sample collection. We randomly collected 4 seedlings, 4 rosettes and 4 fruiting plants around each of ten intersections of the gridlines but no further than 5 m away. Seedlings and rosettes were transported to the Experimental garden where they were grown to maturity and subjected to allozyme electrophoresis. From each fruiting plant, a single young expanded leaf was collected in the field, kept in a cool box and transported to the laboratory for subsequent analysis. Soil samples of summer seed banks were taken and processed in the same way as in the winter seed bank.

In total, 1986 samples were analysed, i.e., 759 in *Carduus acanthoides*, 561 in *Amaranthus retroflexus* and 666 in *Pastinaca sativa*. All samples were analysed by gel electrophoresis using selected enzymes (for methodological details of analysing *Carduus acanthoides*, see [46], for *Amaranthus retroflexus,* see [45]). Data for *Pastinaca sativa* have not yet been published. We have used the following enzymatic systems: Lap, Shdh, Nadh-Dh, 6-Pgdh-1Aat-1, Aat-2, Pgm and Est, using the same methodology as in Mandák *et al*. [45], [46]. For a summary of the enzymatic systems used, see Table 3.

### Statistical Analysis

To estimate genetic diversity and genetic structure, a locus was considered polymorphic if the frequency of the most common allele did not exceed 0.95. Genetic diversity parameters, i.e. allelic richness (*R*_S_), observed heterozygosity (*H*_O_) and gene diversity (*H*_S_) were estimated using the software FSTAT [47]. Population structure was analysed using *F* statistics [48] following the method of Weir and Cockerham [49] by FSTAT. Wright’s *F* statistics [48] include *f*(*F*_IS_), which represents the inbreeding index due to non-random mating within populations, and *θ*(*F*_ST_), which represents the fixation index due to population subdivision. This method allowed us to test whether these *F* statistics were significantly different from zero (determined by 5000 bootstrap replicates). Probability values for differences between various life history stages are given for two-sided t-tests after 10 000 permutations. All analyses were performed using FSTAT.

To test for differences in allelic composition among stages and populations, we ran an exact test of population differentiation [50] with the programme TFPGA [51], using 1000 dememorization steps, 20 batches and 2000 permutations per batch. This test was applied to a contingency table (Fisher’s R×C test) and a Markov chain Monte Carlo approach to determine whether significant differences in allele frequencies exist between stages or populations.

Genetic variation at the level of populations and stages was investigated with a nested analysis of molecular variance (stages nested within populations; AMOVA [52]). Levels of genetic differentiation were measured by *F*_CT_, *F*_SC_, and *F*_ST_, referring to the differentiation among populations, among stages within populations and within stages, respectively. Levels of significance were determined by computing 1000 random permutation replicates.

### Permissions

No specific permits were required for the described field studies, which took place on localities with public right-of-way and did not involve endangered or protected species.

## Supporting Information

Table S1 **Allele frequencies of** ***Amaranthus retroflexus*** **.** Allele frequencies at six loci in four populations and four life history stages of *Amaranthus retroflexus* are given (.pdf).
(PDF)

Table S2 **Allele frequencies of** ***Carduus acanthoides*** **.** Allele frequencies at eight loci in four populations and life history stages of *Carduus acanthoides* are given (.pdf).
(PDF)

Table S3 **Allele frequencies of** ***Pastinaca sativa*** **.** Allele frequencies at eight loci in four populations and five life history stages of *Pastinaca sativa* are given (.pdf).
(PDF)
