# Supplementary material for: Can Soil Seed Banks Serve as Genetic Memory? A Study of Three Species with Contrasting Life History Strategies
Source: PLoS One. 2012 Nov 21;7(11):e49471. doi: 10.1371/journal.pone.0049471 (PMC3504043; doi:10.1371/journal.pone.0049471)
Supplement: Table S1 — Allele frequencies of Amaranthus retroflexus . Allele frequencies at six loci in four populations and four life history stages of Amaranthus retroflexus are given (.pdf). (PDF) [file pone.0049471.s001.pdf]

Table S1 Allele frequencies at six loci in four populations and four life history stages of *Amaranthus retroflexus* calculated by FSTAT [34]. Loc = locality: B – Besno, Po – Pocedelice, Ps – Psov, V – Vojnice; WSB – winter seed bank, SEED – seedlings, SSB – summer seed bank, FP – fruiting plants. Observed and expected heterozygosities as well as departures from the expected Hardy–Weinberg heterozygosity frequencies were computed using Arlequin 3.1 [39]; – allele absent in sample. Statistically significant departures from the expected Hardy–Weinberg heterozygosity frequencies are indicated in bold.

			Allele frequency								Heterozygosity		
Locus	Loc	Stage	Allele 1	Allele 2	Allele 3	Allele 4	Allele 5	Allele 6	Allele 7	Allele 8	Obs	Exp	P
6–Pgdh–1	B	WSB	0	1	–	–	–	–	–	–			
		SEED	0	1	–	–	–	–	–	–			
		SSB	0	1	–	–	–	–	–	–			
		FP	0	1	–	–	–	–	–	–			
	Po	WSB	0	1	–	–	–	–	–	–			
		SEED	0	1	–	–	–	–	–	–			
		SSB	0	1	–	–	–	–	–	–			
		FP	0	1	–	–	–	–	–	–			
	Ps	WSB	0	1	–	–	–	–	–	–			
		SEED	0	1	–	–	–	–	–	–			
		SSB	0	1	–	–	–	–	–	–			
		FP	0	1	–	–	–	–	–	–			
	V	WSB	0	1	–	–	–	–	–	–			
		SEED	0.013	0.988	–	–	–	–	–	–	0.025	0.025	1.000
		SSB	0	1	–	–	–	–	–	–			
		FP	0	1	–	–	–	–	–	–			
6–Pgdh–2	B	WSB	1	–	–	–	–	–	–	–			
		SEED	1	–	–	–	–	–	–	–			

		SSB	1	–	–	–	–	–	–	–			
		FP	1	–	–	–	–	–	–	–			
	Po	WSB	1	–	–	–	–	–	–	–			
		SEED	1	–	–	–	–	–	–	–			
		SSB	1	–	–	–	–	–	–	–			
		FP	1	–	–	–	–	–	–	–			
	Ps	WSB	1	–	–	–	–	–	–	–			
		SEED	1	–	–	–	–	–	–	–			
		SSB	1	–	–	–	–	–	–	–			
		FP	1	–	–	–	–	–	–	–			
	V	WSB	1	–	–	–	–	–	–	–			
		SEED	1	–	–	–	–	–	–	–			
		SSB	1	–	–	–	–	–	–	–			
		FP	1	–	–	–	–	–	–	–			
Pgm	B	WSB	0	1	–	–	–	–	–	–			
		SEED	0	1	–	–	–	–	–	–			
		SSB	0	1	–	–	–	–	–	–			
		FP	0.038	0.963	–	–	–	–	–	–	0.025	0.073	**0.038**
	Po	WSB	0.233	0.767	–	–	–	–	–	–	0.667	0.370	**0.005**
		SEED	0.250	0.750	–	–	–	–	–	–	0.050	0.380	**10^{-4}**
		SSB	0.500	0.500	–	–	–	–	–	–	0.143	0.538	0.118
		FP	0.188	0.813	–	–	–	–	–	–	0.025	0.309	**10^{-4}**
	Ps	WSB	0.675	0.325	–	–	–	–	–	–	0.100	0.444	**10^{-4}**
		SEED	0.550	0.450	–	–	–	–	–	–	0.250	0.501	**0.002**
		SSB	0.600	0.400	–	–	–	–	–	–	0.150	0.486	**10^{-4}**
		FP	0.350	0.650	–	–	–	–	–	–	0.200	0.461	**10^{-4}**
	V	WSB	0.658	0.342	–	–	–	–	–	–	0.158	0.462	**0.006**
		SEED	0.713	0.288	–	–	–	–	–	–	0.125	0.415	**10^{-4}**
		SSB	0.838	0.163	–	–	–	–	–	–	0.125	0.276	**0.004**
		FP	0.688	0.313	–	–	–	–	–	–	0.175	0.435	**10^{-4}**
Dia 2	B	WSB	0.475	0.525	–	–	–	–	–	–	0.100	0.505	**10^{-4}**

		SEED	0.613	0.388	–	–	–	–	–	–	0.075	0.481	**10^{-4}**
		SSB	0.663	0.338	–	–	–	–	–	–	0.025	0.453	**10^{-4}**
		FP	0.475	0.525	–	–	–	–	–	–	0.100	0.505	**10^{-4}**
	Po	WSB	0.733	0.267	–	–	–	–	–	–	0.267	0.405	0.226
		SEED	0.663	0.338	–	–	–	–	–	–	0.175	0.453	**10^{-4}**
		SSB	0.786	0.214	–	–	–	–	–	–	0.143	0.363	0.231
		FP	0.850	0.150	–	–	–	–	–	–	0.050	0.258	**10^{-4}**
	Ps	WSB	0.750	0.250	–	–	–	–	–	–	0.100	0.380	**10^{-4}**
		SEED	0.613	0.388	–	–	–	–	–	–	0.125	0.481	**10^{-4}**
		SSB	0.625	0.375	–	–	–	–	–	–	0.250	0.475	**0.006**
		FP	0.638	0.363	–	–	–	–	–	–	0.225	0.468	**0.002**
	V	WSB	0.658	0.342	–	–	–	–	–	–	0.263	0.462	0.116
		SEED	0.663	0.338	–	–	–	–	–	–	0.125	0.453	**10^{-4}**
		SSB	0.750	0.250	–	–	–	–	–	–	0.200	0.380	**0.005**
		FP	0.775	0.225	–	–	–	–	–	–	0.150	0.353	**0.001**
Est 1	B	WSB	0	0	0	0.050	0.150	0	0.725	0.075	0.100	0.449	**10^{-4}**
		SEED	0	0	0	0	0.038	0.075	0.875	0.013	0.025	0.230	**10^{-4}**
		SSB	0	0	0.025	0.125	0	0.750	0	0.100	0.250	0.416	**10^{-4}**
		FP	0	0	0	0.188	0.250	0	0.563	0	0.275	0.593	**10^{-4}**
	Po	WSB	0.067	0	0	0.200	0.133	0.067	0.533	0	0.133	0.671	**10^{-4}**
		SEED	0.075	0	0.238	0.113	0.250	0	0.300	0.025	0.550	0.782	**10^{-4}**
		SSB	0.143	0	0.286	0.214	0.071	0.286	0	0	0.571	0.824	**0.033**
		FP	0	0	0	0.150	0.475	0.300	0.013	0.063	0.500	0.666	**10^{-4}**
	Ps	WSB	0	0	0.050	0.363	0.400	0.100	0.050	0.038	0.325	0.701	**10^{-4}**
		SEED	0	0.013	0.063	0.338	0.463	0.063	0	0.063	0.475	0.669	**10^{-4}**
		SSB	0.125	0	0.088	0.125	0.613	0.025	0	0.025	0.300	0.592	**10^{-4}**
		FP	0	0	0.050	0.513	0.250	0	0.075	0.113	0.600	0.662	**0.009**
	V	WSB	0.105	0	0.579	0.105	0.184	0	0	0.026	0.421	0.624	**10^{-4}**
		SEED	0.013	0	0.450	0.063	0.438	0	0.025	0.013	0.575	0.609	**10^{-4}**
		SSB	0.088	0	0.600	0.088	0.213	0	0	0.013	0.375	0.587	**10^{-4}**
		FP	0	0	0.013	0.025	0.925	0	0.013	0.025	0.075	0.145	**0.003**

Adh	B	WSB	1	–	–	–	–	–	–	–			
		SEED	1	–	–	–	–	–	–	–			
		SSB	1	–	–	–	–	–	–	–			
		FP	1	–	–	–	–	–	–	–			
	Po	WSB	1	–	–	–	–	–	–	–			
		SEED	1	–	–	–	–	–	–	–			
		SSB	1	–	–	–	–	–	–	–			
		FP	1	–	–	–	–	–	–	–			
	Ps	WSB	1	–	–	–	–	–	–	–			
		SEED	1	–	–	–	–	–	–	–			
		SSB	1	–	–	–	–	–	–	–			
		FP	1	–	–	–	–	–	–	–			
	V	WSB	1	–	–	–	–	–	–	–			
		SEED	1	–	–	–	–	–	–	–			
		SSB	1	–	–	–	–	–	–	–			
		FP	1	–	–	–	–	–	–	–			
