# Supplementary material for: Can Soil Seed Banks Serve as Genetic Memory? A Study of Three Species with Contrasting Life History Strategies
Source: PLoS One. 2012 Nov 21;7(11):e49471. doi: 10.1371/journal.pone.0049471 (PMC3504043; doi:10.1371/journal.pone.0049471)
Supplement: Table S2 — Allele frequencies of Carduus acanthoides . Allele frequencies at eight loci in four populations and life history stages of Carduus acanthoides are given (.pdf). (PDF) [file pone.0049471.s002.pdf]

Table S2 Allele frequencies at eight polymorphic loci in four populations and life history stages of *Carduus acanthoides* calculated by FSTAT [34]. Loc = locality: B – Bustehrad, D – Dobris, O – Orech, V – Velen; WSB – winter seed bank, ROS – rosette, SSB – summer seed bank, FP – fruiting plants. Observed and expected heterozygosities as well as departures from the expected Hardy–Weinberg heterozygosity frequencies performed with Arlequin 3.1 [39]; – allele absent in sample. Statistically significant departures from the expected Hardy–Weinberg heterozygosity frequencies are indicated in bold.

			Allele frequency					Heterozygosity		
Locus	Loc	Stage	Allele 1	Allele 2	Allele 3	Allele 4	Allele 5	Obs	Exp	P
Lap	B	WSB	0.113	0.625	0.263	–	–	0.550	0.531	0.218
		ROS	0.063	0.650	0.288	–	–	0.550	0.497	0.077
		SSB	0.175	0.563	0.263	–	–	0.650	0.591	0.867
		FP	0.075	0.638	0.288	–	–	0.400	0.512	**0.042**
	D	WSB	0.294	0.606	0.100	–	–	0.550	0.540	**0.045**
		RO	0.263	0.563	0.175	–	–	0.600	0.591	0.280
		SSB	0.225	0.650	0.125	–	–	0.625	0.518	0.352
		FP	0.275	0.588	0.138	–	–	0.450	0.567	**0.019**
	O	WSB	0.014	0.740	0.247	–	–	0.384	0.395	**0.013**
		RO	0	0.688	0.313	–	–	0.425	0.435	1.000
		SSB	0	0.750	0.250	–	–	0.500	0.409	1.000
		FP	0	0.713	0.288	–	–	0.475	0.415	0.454
	V	WSB	0.013	0.863	0.125	–	–	0.200	0.242	0.169
		RO	0.013	0.900	0.088	–	–	0.100	0.184	**0.032**
		SSB	0.013	0.888	0.100	–	–	0.225	0.205	1.000
		FP	0.013	0.888	0.100	–	–	0.175	0.205	0.394
SHDH	B	WSB	0.013	0.519	0.263	0.206	–	0.388	0.623	**0.000**
		ROS	0	0.413	0.400	0.188	–	0.475	0.643	**0.000**
		SSB	0	0.795	0.103	0.103	–	0.205	0.352	**0.005**
		FP	0	0.563	0.225	0.213	–	0.475	0.595	**0.000**
	D	WSB	0	0.631	0.169	0.200	–	0.188	0.536	**0.000**
		RO	0	0.400	0.363	0.238	–	0.300	0.660	**0.000**
		SSB	0	0.575	0.325	0.100	–	0.150	0.561	**0.000**
		FP	0	0.450	0.300	0.250	–	0.450	0.653	**0.000**
	O	WSB	0.014	0.397	0.185	0.404	–	0.466	0.649	**0.000**
		RO	0	0.463	0.025	0.513	–	0.225	0.529	**0.000**
		SSB	0	0.833	0.167	0	–	0.000	0.303	0.091
		FP	0.013	0.425	0.100	0.463	–	0.375	0.603	**0.000**
	V	WSB	0.044	0.700	0.044	0.213	–	0.288	0.464	**0.000**
		RO	0	0.763	0	0.238	–	0.225	0.367	**0.023**
		SSB	0	0.850	0.025	0.125	–	0.250	0.265	0.619
		FP	0.013	0.750	0	0.238	–	0.275	0.386	**0.040**
ADH	B	WSB	0.181	0.819	0	–	–	0.313	0.299	1.000

		ROS	0.113	0.888	0	–	–	0.175	0.202	0.395
		SSB	0.225	0.763	0.013	–	–	0.325	0.372	0.519
		FP	0.250	0.750	0	–	–	0.350	0.380	0.678
	D	WSB	0.081	0.919	0	–	–	0.113	0.150	0.073
		RO	0.088	0.913	0	–	–	0.175	0.162	1.000
		SSB	0.100	0.900	0	–	–	0.200	0.182	1.000
		FP	0.100	0.900	0	–	–	0.200	0.182	1.000
	O	WSB	0.199	0.795	0.007	–	–	0.329	0.332	1.000
		RO	0.163	0.825	0.013	–	–	0.200	0.297	0.073
		SSB	0.167	0.833	0	–	–	0.000	0.303	0.090
		FP	0.150	0.850	0	–	–	0.300	0.258	0.567
	V	WSB	0.194	0.794	0.013	–	–	0.313	0.334	0.669
		RO	0.188	0.788	0.025	–	–	0.375	0.348	1.000
		SSB	0.275	0.725	0	–	–	0.500	0.404	0.228
		FP	0.213	0.775	0.013	–	–	0.250	0.359	0.111
6–PGDH–1	B	WSB	0.788	0.213	–	–	–	0.375	0.337	0.500
		ROS	0.813	0.188	–	–	–	0.275	0.309	0.598
		SSB	0.700	0.300	–	–	–	0.300	0.425	0.125
		FP	0.863	0.138	–	–	–	0.275	0.240	1.000
	D	WSB	0.556	0.444	–	–	–	0.463	0.497	0.650
		RO	0.325	0.675	–	–	–	0.450	0.444	1.000
		SSB	0.463	0.538	–	–	–	0.425	0.503	0.354
		FP	0.538	0.463	–	–	–	0.475	0.503	0.758
	O	WSB	0.589	0.411	–	–	–	0.466	0.487	0.810
		RO	0.588	0.413	–	–	–	0.375	0.491	0.191
		SSB	0.500	0.500	–	–	–	0.667	0.545	1.000
		FP	0.588	0.413	–	–	–	0.525	0.491	0.748
	V	WSB	0.638	0.363	–	–	–	0.450	0.465	0.812
		RO	0.588	0.413	–	–	–	0.375	0.491	0.191
		SSB	0.675	0.325	–	–	–	0.350	0.444	0.275
		FP	0.650	0.350	–	–	–	0.450	0.461	1.000
6–PGDH–1	B	WSB	0.763	0.156	0.081	–	–	0.325	0.390	**0.006**
		ROS	0.700	0.175	0.125	–	–	0.475	0.470	0.623
		SSB	0.763	0.138	0.100	–	–	0.450	0.395	1.000
		FP	0.750	0.175	0.075	–	–	0.375	0.406	0.768
	D	WSB	0.913	0.031	0.056	–	–	0.175	0.164	1.000
		RO	0.925	0.038	0.038	–	–	0.150	0.143	1.000
		SSB	0.913	0.013	0.075	–	–	0.175	.0.164	1.000
		FP	0.938	0.038	0.025	–	–	0.100	0.121	0.122
	O	WSB	0.952	0.048	0	–	–	0.068	0.092	0.140
		RO	0.950	0.050	0	–	–	0.100	0.096	1.000
		SSB	1.000	0	0	–	–			
		FP	0.900	0.100	0	–	–	0.200	0.182	1.000
	V	WSB	0.744	0.044	0.213	–	–	0.375	0.402	0.426
		RO	0.763	0.013	0.225	–	–	0.450	0.372	**0.045**
		SSB	0.838	0.013	0.150	–	–	0.225	0.279	0.316
		FP	0.763	0.013	0.225	–	–	0.300	0.372	0.089
AAT–1	B	WSB	0.013	0.025	0.962	–	–	0.051	0.074	**0.045**

		ROS	0.013	0.038	0.950	–	–	0.100	0.097	1.000
		SSB	0	0.013	0.987	–	–	0.026	0.026	1.000
		FP	0.063	0.038	0.900	–	–	0.050	0.187	0.000
	D	WSB	0.006	0.032	0.962	–	–	0.051	0.074	0.093
		RO	0.038	0.050	0.913	–	–	0.175	0.166	1.000
		SSB	0.013	0.025	0.963	–	–	0.075	0.074	1.000
		FP	0.100	0.075	0.825	–	–	0.300	0.308	0.311
	O	WSB	0	0	1.000	–	–			
		RO	0	0	1.000	–	–			
		SSB	0	0	1.000	–	–			
		FP	0.013	0	0.988	–	–	0.025	0.250	1.000
	V	WSB	0.006	0.013	0.981	–	–	0.013	0.037	**0.006**
		RO	0	0	1.000	–	–			
		SSB	0.013	0	0.988	–	–	0.025	0.025	1.000
		FP	0	0.013	0.988	–	–	0.025	0.025	1.000
AAT–2	B	WSB	0.669	0.331	–	–	–	0.438	0.446	1.000
		ROS	0.588	0.413	–	–	–	0.425	0.491	0.515
		SSB	0.613	0.388	–	–	–	0.475	0.481	1.000
		FP	0.488	0.513	–	–	–	0.625	0.506	0.204
	D	WSB	0.650	0.350	–	–	–	0.475	0.458	0.808
		RO	0.513	0.488	–	–	–	0.425	0.506	0.355
		SSB	0.663	0.338	–	–	–	0.525	0.453	0.477
		FP	0.563	0.438	–	–	–	0.425	0.498	0.519
	O	WSB	0.507	0.493	–	–	–	0.521	0.503	0.816
		RO	0.575	0.425	–	–	–	0.300	0.495	**0.021**
		SSB	0.917	0.083	–	–	–	0.167	0.167	1.000
		FP	0.600	0.400	–	–	–	0.650	0.486	**0.047**
	V	WSB	0.656	0.344	–	–	–	0.438	0.454	0.806
		RO	0.475	0.525	–	–	–	0.550	0.505	0.752
		SSB	0.550	0.450	–	–	–	0.450	0.501	0.537
		FP	0.613	0.388	–	–	–	0.575	0.481	0.317
DIA–1	B	WSB	0	0.238	0	0.638	0.125	0.450	0.525	0.259
		ROS	0	0.300	0.013	0.525	0.163	0.550	0.616	0.594
		SSB	0	0.250	0	0.613	0.138	0.450	0.550	0.352
		FP	0.050	0.263	0.025	0.525	0.138	0.725	0.641	0.058
	D	WSB	0.013	0.494	0	0.319	0.175	0.663	0.628	0.099
		RO	0.100	0.550	0	0.288	0.063	0.800	0.609	0.010
		SSB	0	0.513	0	0.363	0.125	0.650	0.598	0.594
		FP	0.025	0.575	0	0.300	0.100	0.500	0.576	0.263
	O	WSB	0	0.685	0	0.260	0.055	0.438	0.463	0.880
		RO	0.038	0.588	0	0.225	0.150	0.575	0.588	0.668
		SSB	0	0.667	0	0.333	0	0.667	0.485	1.000
		FP	0.063	0.575	0	0.250	0.113	0.650	0.598	0.085
	V	WSB	0.013	0.356	0.013	0.581	0.038	0.538	0.537	0.604
		RO	0	0.413	0	0.563	0.025	0.425	0.519	0.293
		SSB	0	0.413	0	0.563	0.025	0.700	0.519	**0.037**
		FP	0	0.450	0	0.513	0.038	0.675	0.540	0.094
