# Supplementary material for: Can Soil Seed Banks Serve as Genetic Memory? A Study of Three Species with Contrasting Life History Strategies
Source: PLoS One. 2012 Nov 21;7(11):e49471. doi: 10.1371/journal.pone.0049471 (PMC3504043; doi:10.1371/journal.pone.0049471)
Supplement: Table S3 — Allele frequencies of Pastinaca sativa . Allele frequencies at eight loci in four populations and five life history stages of Pastinaca sativa are given (.pdf). (PDF) [file pone.0049471.s003.pdf]

Table S3 Allele frequencies at eight loci in four populations and five life history stages of *Pastinaca sativa* calculated by FSTAT [34]. Loc = locality: DB – Dobre Pole, O – Ostopovice, S – Sedlec, Z – Zidneves; WSB – winter seed bank, SEED – seedlings, RO – rosettes, SSB – summer seed bank, FP – fruiting plants. Observed and expected heterozygosities as well as departures from the expected Hardy–Weinberg heterozygosity frequencies performed with Arlequin 3.1 [39]; – allele absent in sample. Statistically significant departures from the expected Hardy–Weinberg heterozygosity frequencies are indicated in bold.

			Allele frequency							Heterozygosity		
Locus	Loc	Stage	Allele 1	Allele 2	Allele 3	Allele 4	Allele 5	Allele 6	Allele 7	Obs	Exp	P
Lap	DP	WSB	0	0.038	0.088	0.688	0.013	0.175	0	0.375	0.494	0.052
		SEED	0.050	0.038	0.175	0.588	0.038	0.113	0	0.625	0.614	0.921
		RO	0.013	0.063	0.163	0.625	0.013	0.125	0	0.400	0.570	**0.003**
		SSB	0	0	0.067	0.767	0	0.167	0	0.333	0.393	0.078
		FP	0.025	0.025	0.075	0.788	0	0.088	0	0.375	0.370	0.632
	O	WSB	0	0.038	0.350	0.500	0	0.113	0	0.450	0.621	**0.014**
		SEED	0.013	0	0.250	0.600	0	0.138	0	0.500	0.566	0.259
		RO	0	0.015	0.258	0.636	0	0.091	0	0.515	0.528	0.883
		SSB	0	0	0.400	0.600	0	0	0	0.000	0.533	**0.047**
		FP	0	0	0.138	0.713	0	0.150	0	0.400	0.457	**0.001**
	Z	WSB	0.063	0.013	0.338	0.563	0	0.025	0	0.425	0.572	**0.040**
		SEED	0.075	0	0.288	0.638	0	0	0	0.425	0.512	0.443
		RO	0.088	0	0.350	0.563	0	0	0	0.225	0.560	$\mathbf{10^{-4}}$
		SSB	0.065	0	0.217	0.696	0	0.022	0	0.174	0.474	$\mathbf{10^{-4}}$
		FP	0.088	0.025	0.450	0.438	0	0	0	0.300	0.605	$\mathbf{10^{-4}}$
	S	WSB	0.019	0.019	0.058	0.692	0	0.173	0.038	0.462	0.495	0.085
		SEED	0	0.063	0.125	0.613	0	0.175	0.025	0.600	0.581	0.859

		RO	0	0.075	0.250	0.425	0	0.238	0.013	0.425	0.703	**10^{-4}**
		SSB	0	0	0	0.625	0	0.375	0	0.750	0.536	1.000
		FP	0	0.063	0.113	0.550	0	0.250	0.025	0.500	0.626	0.229
Shdh	DP	WSB	0.013	0.175	0	0.813	0	–	–	0.275	0.313	0.653
		SEED	0.025	0.200	0	0.775	0	–	–	0.350	0.363	0.780
		RO	0.013	0.244	0	0.744	0	–	–	0.436	0.393	0.220
		SSB	0	0.167	0	0.833	0	–	–	0.333	0.287	1.000
		FP	0	0.175	0	0.813	0.013	–	–	0.325	0.313	1.000
	O	WSB	0.025	0.213	0.013	0.750	0	–	–	0.375	0.397	0.618
		SEED	0.013	0.263	0	0.713	0.013	–	–	0.450	0.428	0.526
		RO	0.030	0.288	0	0.636	0.045	–	–	0.576	0.517	0.061
		SSB	0	0.500	0	0.500	0	–	–	0.600	0.556	1.000
		FP	0.038	0.325	0	0.638	0	–	–	0.475	0.493	0.068
	Z	WSB	0.013	0.275	0	0.713	0	–	–	0.375	0.422	0.599
		SEED	0.013	0.450	0	0.538	0	–	–	0.300	0.515	**0.006**
		RO	0	0.450	0	0.550	0	–	–	0.400	0.501	0.217
		SSB	0	0.500	0	0.500	0	–	–	0.478	0.511	1.000
		FP	0.013	0.438	0	0.550	0	–	–	0.475	0.512	0.620
	S	WSB	0.019	0.462	0	0.519	0	–	–	0.462	0.527	0.546
		SEED	0.050	0.425	0.013	0.513	0	–	–	0.450	0.561	0.333
		RO	0	0.450	0.025	0.513	0.013	–	–	0.550	0.541	0.642
		SSB	0	0.375	0	0.625	0	–	–	0.250	0.536	0.429
		FP	0.013	0.438	0.025	0.525	0	–	–	0.625	0.539	0.636
Nadh–Dh	DP	WSB	0.013	0.988	–	–	–	–	–	0.025	0.025	1.000
		SEED	0.025	0.975	–	–	–	–	–	0.050	0.049	1.000
		RO	0.050	0.950	–	–	–	–	–	0.100	0.096	1.000
		SSB	0.033	0.967	–	–	–	–	–	0.067	0.067	1.000
		FP	0.063	0.938	–	–	–	–	–	0.075	0.119	0.124
	O	WSB	0.150	0.850	–	–	–	–	–	0.100	0.258	**0.001**
		SEED	0.038	0.963	–	–	–	–	–	0.075	0.073	1.000

		RO	0.045	0.955	–	–	–	–	–	0.030	0.088	**0.047**
		SSB	0.100	0.900	–	–	–	–	–	0.200	0.200	1.000
		FP	0.025	0.975	–	–	–	–	–	0.050	0.049	1.000
	Z	WSB	0.150	0.850	–	–	–	–	–	0.150	0.258	**0.026**
		SEED	0.100	0.900	–	–	–	–	–	0.200	0.182	1.000
		RO	0.075	0.925	–	–	–	–	–	0.150	0.141	1.000
		SSB	0.174	0.826	–	–	–	–	–	0.348	0.294	1.000
		FP	0.113	0.888	–	–	–	–	–	0.225	0.202	1.000
	S	WSB	0.019	0.981	–	–	–	–	–	0.038	0.038	1.000
		SEED	0.013	0.988	–	–	–	–	–	0.025	0.025	1.000
		RO	0.013	0.988	–	–	–	–	–	0.025	0.025	1.000
		SSB	0	1	–	–	–	–	–			
		FP	0.013	0.988	–	–	–	–	–	0.025	0.025	1.000
6–Pgdh–1	DP	WSB	0.488	0.513	–	–	–	–	–	0.975	0.506	$\mathbf{10^{-4}}$
		SEED	0.500	0.500	–	–	–	–	–	1.000	0.506	$\mathbf{10^{-4}}$
		RO	0.500	0.500	–	–	–	–	–	1.000	0.506	$\mathbf{10^{-4}}$
		SSB	0.500	0.500	–	–	–	–	–	1.000	0.517	$\mathbf{10^{-4}}$
		FP	0.500	0.500	–	–	–	–	–	1.000	0.506	$\mathbf{10^{-4}}$
	O	WSB	0.500	0.500	–	–	–	–	–	1.000	0.506	$\mathbf{10^{-4}}$
		SEED	0.500	0.500	–	–	–	–	–	1.000	0.506	$\mathbf{10^{-4}}$
		RO	0.500	0.500	–	–	–	–	–	1.000	0.508	$\mathbf{10^{-4}}$
		SSB	0.500	0.500	–	–	–	–	–	1.000	0.556	0.127
		FP	0.500	0.500	–	–	–	–	–	1.000	0.506	$\mathbf{10^{-4}}$
	Z	WSB	0.488	0.513	–	–	–	–	–	0.975	0.506	$\mathbf{10^{-4}}$
		SEED	0.500	0.500	–	–	–	–	–	1.000	0.506	$\mathbf{10^{-4}}$
		RO	0.500	0.500	–	–	–	–	–	1.000	0.506	$\mathbf{10^{-4}}$
		SSB	0.500	0.500	–	–	–	–	–	1.000	0.511	$\mathbf{10^{-4}}$
		FP	0.488	0.513	–	–	–	–	–	0.975	0.506	$\mathbf{10^{-4}}$
	S	WSB	0.500	0.500	–	–	–	–	–	1.000	0.510	$\mathbf{10^{-4}}$
		SEED	0.500	0.500	–	–	–	–	–	1.000	0.506	$\mathbf{10^{-4}}$

		RO	0.500	0.500	–	–	–	–	–	1.000	0.506	**10^{-4}**
		SSB	0.500	0.500	–	–	–	–	–	1.000	0.571	0.314
		FP	0.488	0.513	–	–	–	–	–	0.975	0.506	**10^{-4}**
Aat–1	DP	WSB	0	0.038	0.950	0.013	–	–	–	0.100	0.097	1.000
		SEED	0.013	0.013	0.938	0.038	–	–	–	0.125	0.121	1.000
		RO	0	0.013	0.975	0.013	–	–	–	0.050	0.050	1.000
		SSB	0	0	1	0	–	–	–			
		FP	0	0.025	0.925	0.050	–	–	–	0.150	0.143	1.000
	O	WSB	0	0	0.987	0.013	–	–	–	0.026	0.026	1.000
		SEED	0	0	1	0	–	–	–			
		RO	0	0	1	0	–	–	–			
		SSB	0	0	1	0	–	–	–			
		FP	0	0	1	0		–	–			
	Z	WSB	0	0	1	0	–	–	–			
		SEED	0	0	1	0	–	–	–			
		RO	0	0	1	0	–	–	–			
		SSB	0	0	1	0	–	–	–			
		FP	0	0	1	0	–	–	–			
	S	WSB	0.058	0.058	0.827	0.058	–	–	–	0.231	0.312	0.078
		SEED	0	0.088	0.850	0.063	–	–	–	0.250	0.269	0.130
		RO	0	0.113	0.813	0.075	–	–	–	0.250	0.326	0.085
		SSB	0	0	1	0	–	–	–			
		FP	0	0.088	0.875	0.038	–	–	–	0.225	0.228	0.469
Aat–2	DP	WSB	0.025	0.975	–	–	–	–	–	0.050	0.049	1.000
		SEED	0.025	0.975	–	–	–	–	–	0.050	0.049	1.000
		RO	0.013	0.988	–	–	–	–	–	0.025	0.025	1.000
		SSB	0.100	0.900	–	–	–	–	–	0.200	0.186	1.000
		FP	0.038	0.963	–	–	–	–	–	0.075	0.073	1.000
	O	WSB	0.050	0.950	–	–	–	–	–	0.050	0.096	0.075
		SEED	0.013	0.988	–	–	–	–	–	0.025	0.025	1.000

		RO	0.030	0.970	–	–	–	–	–	0.061	0.060	1.000
		SSB	0	1	–	–	–	–	–			
		FP	0	1	–	–	–	–	–			
	Z	WSB	0	1	–	–	–	–	–			
		SEED	0	1	–	–	–	–	–			
		RO	0	1	–	–	–	–	–			
		SSB	0	1	–	–	–	–	–			
		FP	0	1	–	–	–	–	–			
	S	WSB	0.019	0.981	–	–	–	–	–	0.038	0.038	1.000
		SEED	0.038	0.963	–	–	–	–	–	0.075	0.073	1.000
		RO	0.013	0.988	–	–	–	–	–	0.025	0.025	1.000
		SSB	0.125	0.875	–	–	–	–	–	0.250	0.250	1.000
		FP	0.025	0.975	–	–	–	–	–	0.050	0.049	1.000
Pgm	DP	WSB	0	0.713	0.288	–	–	–	–	0.075	0.415	**10^{-4}**
		SEED	0	0.850	0.150	–	–	–	–	0.100	0.258	**0.002**
		RO	0	0.888	0.113	–	–	–	–	0.125	0.202	0.056
		SSB	0	0.967	0.033	–	–	–	–	0.067	0.067	1.000
		FP	0	0.788	0.213	–	–	–	–	0.075	0.339	**10^{-4}**
	O	WSB	0	0.988	0.013	–	–	–	–	0.025	0.025	1.000
		SEED	0	0.950	0.050	–	–	–	–	0.050	0.096	0.075
		RO	0.015	0.909	0.076	–	–	–	–	0.061	0.170	**0.004**
		SSB	0	0.900	0.100	–	–	–	–	0.200	0.200	1.000
		FP	0	0.925	0.075	–	–	–	–	0.100	0.141	0.182
	Z	WSB	0	1	0	–	–	–	–			
		SEED	0	1	0	–	–	–	–			
		RO	0	1	0	–	–	–	–			
		SSB	0	1	0	–	–	–	–			
		FP	0	1	0	–	–	–	–			
	S	WSB	0	0.404	0.596	–	–	–	–	0.038	0.491	**10^{-4}**
		SEED	0	0.925	0.075	–	–	–	–	0.050	0.141	**0.007**

		RO	0	0.925	0.075	–	–	–	–	0.050	0.141	**0.007**
		SSB	0	1	0	–	–	–	–			
		FP	0	0.888	0.113	–	–	–	–	0.025	0.202	**10^{-4}**
Est	DP	WSB	0	0	0.713	0.288	0	–	–	0.375	0.415	0.699
		SEED	0	0	0.850	0.150	0	–	–	0.100	0.258	**0.001**
		RO	0	0	0.813	0.188	0	–	–	0.225	0.309	0.110
		SSB	0	0	0.867	0.133	0	–	–	0.133	0.239	0.204
		FP	0.038	0.013	0.838	0.088	0.025	–	–	0.150	0.292	**0.001**
	O	WSB	0	0	0.950	0.050	0	–	–	0.100	0.096	1.000
		SEED	0	0	0.950	0.050	0	–	–	0.100	0.096	1.000
		RO	0	0	0.833	0.167	0	–	–	0.152	0.282	**0.024**
		SSB	0	0	1	0	0	–	–			
		FP	0.038	0.013	0.863	0.088	0	–	–	0.250	0.250	0.231
	Z	WSB	0	0.025	0.888	0.088	0	–	–	0.200	0.207	0.199
		SEED	0	0.025	0.875	0.100	0	–	–	0.100	0.227	**0.001**
		RO	0	0.088	0.813	0.075	0.025	–	–	0.175	0.330	**0.001**
		SSB	0	0.022	0.891	0.087	0	–	–	0.217	0.202	1.000
		FP	0	0.025	0.788	0.188	0	–	–	0.200	0.348	**0.010**
	S	WSB	0	0	0.673	0.327	0	–	–	0.500	0.449	0.671
		SEED	0	0	0.800	0.163	0.038	–	–	0.350	0.336	1.000
		RO	0	0	0.913	0.075	0.013	–	–	0.125	0.164	0.251
		SSB	0	0	1	0	0	–	–			
		FP	0	0	0.850	0.150	0	–	–	0.250	0.258	1.000
